# DIF-1 induces the basal disc of the *Dictyostelium* fruiting body

**DOI:** 10.1016/j.ydbio.2008.02.036

**Published:** 2008-05-15

**Authors:** Tamao Saito, Atsushi Kato, Robert R. Kay

**Affiliations:** a Department of Biological Sciences, Faculty of Science, Hokkaido University, Sapporo 060-0810, Japan; b MRC Laboratory of Molecular Biology, Cambridge CB2 0QH, UK

**Keywords:** *Dictyostelium*, DIF-1, Polyketide synthase, Basal disc, Lower cup, Slug migration

## Abstract

The polyketide DIF-1 induces *Dictyostelium* amoebae to form stalk cells in culture. To better define its role in normal development, we examined the phenotype of a mutant blocking the first step of DIF-1 synthesis, which lacks both DIF-1 and its biosynthetic intermediate, dM-DIF-1 (des-methyl-DIF-1). Slugs of this polyketide synthase mutant (*stlB*^−^) are long and thin and rapidly break up, leaving an immotile prespore mass. They have ∼ 30% fewer prestalk cells than their wild-type parent and lack a subset of anterior-like cells, which later form the outer basal disc. This structure is missing from the fruiting body, which perhaps in consequence initiates culmination along the substratum. The lower cup is rudimentary at best and the spore mass, lacking support, slips down the stalk. The *dmtA*^−^ methyltransferase mutant, blocked in the last step of DIF-1 synthesis, resembles the *stlB*^−^ mutant but has delayed tip formation and fewer prestalk-O cells. This difference may be due to accumulation of dM-DIF-1 in the *dmtA*^−^ mutant, since dM-DIF-1 inhibits prestalk-O differentiation. Thus, DIF-1 is required for slug migration and specifies the anterior-like cells forming the basal disc and much of the lower cup; significantly the DIF-1 biosynthetic pathway may supply a second signal - dM-DIF-1.

## Introduction

Developing *Dictyostelium* cells become partitioned into the prestalk and prespore lineages toward the end of aggregation (Kessin, 2001). There appears to be a single prespore lineage producing the spore cells of the mature fruiting body. But in contrast, several distinct prestalk lineages can be recognized, and prestalk cells produce four discrete structures in the fruiting body: the basal disc, the stalk proper, and the upper and lower cups that cradle the spores (Williams, 1997). Only the basal disc and stalk consist of mature stalk cells, the upper and lower cup cells remaining amoeboid even in the mature fruiting body. The specification of these diverse cell types involves both cell signaling and intrinsic biases present in the original growing cells and maintained through early development (Leach et al., 1973; Gomer and Firtel, 1987; Thompson and Kay, 2000a).

Prestalk cells form the anterior region of the migrating slug, but are not confined to it: a population of scattered, non-prespore cells also exists in the posterior prespore zone (Sternfeld and David, 1981). These anterior-like cells express at least some prestalk markers and stain with the vital dye neutral red (Devine and Loomis, 1985; Jermyn and Williams, 1991). Their function is not well understood, but may include transmitting cyclic-AMP signals through the prespore zone and initiating culmination and, in the fruiting body, they form the lower cup and basal disc.

Prestalk and stalk cells can be induced in suitable culture conditions by the chlorinated alkyl phenone DIF-1 (1-(3,5-dichloro-2,6-dihydroxy-4-methoxyphenyl)hexan-1-one) (Morris et al., 1987). DIF-1 accumulates in developing cells from the end of aggregation and is released into the medium, but its function in normal development was initially only inferred from its effects in culture. Here, most notably, DIF-1 rapidly induces the expression of a subset of prestalk genes and represses the expression of all tested prespore genes (Kay and Jermyn, 1983; Williams et al., 1987; Early and Williams, 1988; Fosnaugh and Loomis, 1991; Kay, 1997). More definitive information on its role in development would be provided by the phenotype of mutants in which DIF-1 synthesis is specifically blocked. Early work resulted in the isolation of several mutants that produced little DIF, but could still respond to it efficiently. Though these mutants were valuable, their basic lesion remains unknown and their phenotype – arrest at the mound stage of development (Kopachik et al., 1983) – is now known to be misleading. A more directed approach to mutant isolation became possible once the biosynthetic pathway for DIF-1 was elucidated.

**Fig. 1:**
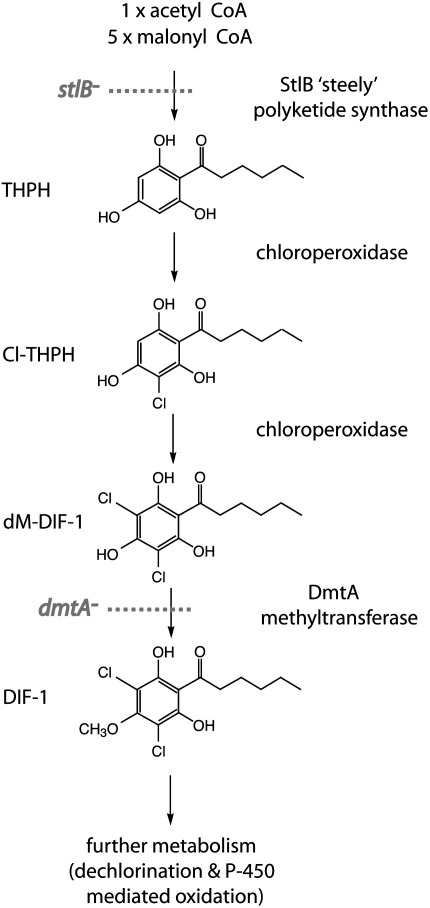
DIF-1 biosynthetic pathway. The polyketide skeleton of DIF-1 is assembled by the StlB, ‘steely’, polyketide synthase, and then decorated by two successive chlorinations (where the chloroperoxidase is only known as an enzymatic activity) and a final methylation by the DmtA methyltransferase (Kay, 1998; Thompson and Kay, 2000b; Austin et al., 2006). DIF-1 is further metabolized by mono-dechlorination to DIF-3 and then by successive hydroxylations/oxidations to yield around 10 further metabolites (Traynor and Kay, 1991; Morandini et al., 1995). These metabolites are all much less active than DIF-1 in the stalk cell bioassay, but may conceivably have other activities *in vivo*.

DIF-1 is made from a 12-carbon polyketide, which is successively chlorinated and methylated to give the final molecule (Fig. 1) (Kay, 1998). The DmtA methyltransferase carrying out the last step in this pathway was identified and eliminated by gene disruption giving a mutant with little if any detectable DIF-1 (Thompson and Kay, 2000b). This mutant produces aberrant fruiting bodies, but surprisingly these contain mature stalk cells. However, a reduced number of prestalk cells are made, with a specific deficit of the prestalk-O (pstO) subtype. A serious uncertainty remains over the full ‘DIFless’ phenotype because subsequent work showed that the mutant retained significant amounts of the blocked intermediates in DIF-1 biosynthesis, desmethyl-DIF-1 (1-(3,5-dichloro-2,4,6-trihydroxyphenyl)hexan-1-one), and Cl-THPH (1-(3-chloro-2,4,6-trihydorxyphenyl)hexan-1-one) (Saito et al., 2006), which are known to have definite stalk-cell inducing activity and might therefore be responsible for inducing at least some of the residual prestalk and stalk cells that do differentiate in the *dmtA*^−^ mutant.

A second set of mutants in the transcription factors DimA, DimB, and MybE are impaired in their response to DIF-1 (Thompson et al., 2004; Huang et al., 2006; Fukuzawa et al., 2006; Zhukovskaya et al., 2006). All develop aberrantly and share phenotypic features with the *dmtA*^−^ mutant. However, they differ in their detailed phenotypes, consistent with each gene having additional functions unrelated to DIF-1 signaling (Foster et al., 2004; Fukuzawa et al., 2006) or only being responsible for part of the transcriptional response to DIF-1 (Huang et al., 2006).

To better define the role of DIF-1 in normal development, we therefore turned to the *stlB* polyketide synthase (PKS) mutant in which DIF-1 biosynthesis is blocked at the start of the pathway, so that no intermediates can accumulate (Austin et al., 2006) (Fig. 1). The *Dictyostelium* genome contains around 40 PKS genes, each encoding a multi-domain protein of more than 2000 amino acids (Eichinger et al., 2005; Zucko et al., 2007). StlB has the novel ‘steely’ domain organization, which it shares with only one other distantly related PKS (StlA). In these proteins, a chalcone synthase domain is fused to the C-terminus of a multi-domain PKS where, in the case of StlB, it makes the phenolic ring of DIF-1. The chalcone synthase domain of StlA has a different specificity, preferentially making pyrones (Austin et al., 2006). It is therefore very likely that StlB is the only source of DIF-1 in the cell and that *stlB*^−^ cells – already known to make less than 5% of wild-type levels of DIF-1 – actually make none at all.

We have analyzed the phenotype of *stlB*^−^ and related mutants in more detail than previously, thus confirming that DIF-1 is required for slug migration and crucially for the formation of the lower cup and basal disc of the fruiting body.

## Materials and methods

### *Dictyostelium* cell culture and development

*Dictyostelium discoideum* strain Ax2 was maintained in HL-5 medium. LacZ transformants of wild-type background were grown in HL-5 medium containing 10 μg/ml of G418, whereas the medium for the *stlB* knockout strain, HM1154, contained both blasticidin (10 μg/ml) and G418 (10 μg/ml), and that of the double knockout mutant of *dmtA* and *stlB* both blasticidin (10 μg/ml) and hygromycin (30 μg/ml). Cells were developed on 1.5% agar (Difco) either unbuffered or with phosphate buffer (2.7 mM Na_2_ HPO_4_/10.7 mM K_2_HPO_4_, pH 6.2). The timing of tip formation was determined using cells plated at 1.5 × 10^6^ cm^− 2^ on 1.8% L28 agar (Oxoid) containing KK2 (16.5 mM KH_2_PO_4_, 3.9 mM K_2_HPO_4_, pH 6.2), 2 mM MgSO_4_, and 0.1 mM CaCl_2_.

### Knockout strain and G418 transformants

The *stlB* gene knockout construct was created by the *in vitro* transposition method as described (Austin et al., 2006). G418 transformants were generated by electroporation and selected at 10 μg/ml G418 in HL-5 medium. For the double knockout mutant of *dmtA* and *stlB*, a transposable hygromycin resistance cassette (a kind gift from Dr J. Williams) was used to create a *stlB*^−^ knockout vector (Abe et al., 2003). Insertion of the transposable hygromycin resistance cassette was confirmed by *Bam*HI digestion and the insertion site mapped by sequencing. The knockout vector was linearized with *Pvu*II and transformed by electroporation into HM1030, *dmtA* null cells. The clones were selected and screened for the disruption of the locus by genomic PCR with various combinations of primers (Supplementary Fig. 1).

### Whole mount lacZ staining, prespore, and Neutral red staining

Exponentially growing cells were harvested and washed with phosphate buffer (pH 6.2). The cell suspension was allowed to develop on a sheet of filter paper (Whatman 50) or nitrocellulose filter (pore size 0.45 μm Millipore) placed on 1.5% non-nutrient agar with or without 100 nM DIF-1. LacZ staining was performed as described (Dingermann et al., 1989). Neutral red staining was performed as described (Siegert and Weijer, 1992) with minor modification, that is the cells were stained with 50 μg/ml neutral red for 10 min. The stained cells were developed on non-nutrient agar plate or on filter.

Prespore/prestalk cell proportions were determined by staining using an antibody prepared against *D. mucoroides* spores (Hayashi and Takeuchi, 1976). Around 20 fully extended first fingers were picked with a micro-needle and disaggregated for 5 min at 22 °C using a pronase/2,3 dimercapto-propanol mix (Takeuchi and Yabuno, 1970) diluted 1:1 with KK2. Cells were then washed, fixed and stained using a fluorescent second antibody. Photographs were taken with a digital camera (Olympus HC-300z/OL) attached to a stereomicroscope (Olympus SZX12) or using a Zeiss photomicroscope.

## Results

### DIF-1 biosynthetic mutants

The backbone of DIF-1 consists of a 12-carbon polyketide produced by the steely B (*stlB*) PKS (Austin et al., 2006). Several independent *stlB* gene disruptants were isolated previously and shown not to produce any detectable DIF-1. Since they had identical phenotypes, one of them, HM1154 (referred to as the PKS null), was selected for detailed study here. After synthesis by StlB, the DIF-1 polyketide is chlorinated and then methylated to produce DIF-1, with methylation catalyzed by the DmtA methyl transferase (Thompson and Kay, 2000b) (Fig. 1). Strains HM1030 and HM1031, the previously described null mutants of *dmtA* (referred to as the methyltransferase null), and a newly created double mutant, in which *stlB* was disrupted in the HM1030 background (strain HM1196, *dmtA*^−^*, stlB*− referred to as the double null), were compared as appropriate. All mutants are in the Ax2 (referred to as wild-type strain) background and all grow normally in axenic medium. The strains used in this study and their genotypes are summarized in Table 1.

### Morphology

**Fig. 2:**
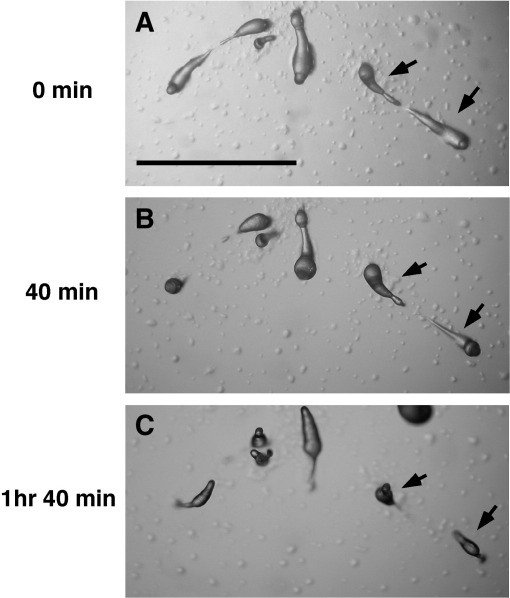
Slugs formed without DIF-1 break up and regulate. Slugs of the PKS null strain were observed from soon after alighting on the agar surface. Note that though the posterior part of the slug does not move, it still regulates and makes a fruiting body. Scale bar indicates 1 mm.

PKS null and methyltransferase null strains develop in a very similar way and the differences from wild-type strain give a common DIFless phenotype. Aggregation is similar to wild-type cells, except that methyltransferase null mutant is delayed in tip formation (Table 2, see below) but clear differences are apparent from tip formation onward. The first fingers of both strains are noticeably long and thin and when these fall to the agar, the resulting slugs break up almost immediately, typically 1/3 of the way back. The anterior portion can migrate off at the same rate as a wild-type slug, whereas the posterior part forms a new tip and generally fruits on the spot, showing that slugs can still regulate in the absence of DIF-1 (Fig. 2).

Fruiting is asynchronous and the final fruiting body is strikingly abnormal. Culmination often initiates along the agar surface, and only after some stalk has been laid down, does it turn upward. The spore mass, instead of being at the top of the stalk, frequently slips down it. These defects combined give a very untidy appearance that is also characteristic of the *dimA*− mutant, which is unable to respond to DIF-1 (Thompson et al., 2004).

These gross developmental defects are corrected by developing either the methyltransferase null or PKS null mutants on agar containing 100 nM DIF-1, confirming that they are due to a lack of DIF-1 and not to an unrelated function of the StlB or DmtA proteins (not shown).

The methyltransferase null strain (HM1030) was previously reported to develop more slowly than wild-type strain (Thompson and Kay, 2000b) and we found a consistent 2 h delay in tip formation compared to wild-type or PKS null strains, which was not rescued by adding DIF-1 to the agar. This was confirmed with strain HM1031, an independent methyltransferase null clone. This might be explained in two ways: either DmtA has an additional function required in aggregation that is unrelated to DIF-1 synthesis, or the accumulation of intermediates in the DIF-1 biosynthetic pathway (Saito et al., 2006) delays tip formation. To distinguish between these possibilities, we examined the double null mutant, which lacks the biosynthetic intermediates, and found that it forms tips with wild-type timing, suggesting that these intermediates delay tip formation (Table 2).

### Prestalk/prespore ratio

The expression kinetics of prestalk and spore marker genes ecmA, ecmB, and spiA were similar between wild-type and the PKS null mutant by RT-PCR (data not shown). The proportion of prestalk cells in the mutants was assessed after disaggregation to single cells using the classical ‘Takeuchi’ antibody against prespore cells (Hayashi and Takeuchi, 1976). Non-staining cells are defined as prestalk cells, and also include anterior-like cells, which are found in the prespore zone. Fully extended first fingers were hand picked with a micro-needle to ensure that all strains were at the same stage and to avoid complications due to the break up and regulation of slugs once they touched down on the agar.

The wild-type strain had a lower proportion of prestalk cells than previously reported for slugs (Hayashi and Takeuchi, 1976, 1981), which may be due to the stage selected or because previously cells that failed to enter developmental structures were also collected (and would be scored as prestalks) or to a strain difference. Both methyltransferase null and PKS null mutants produced only approximately 65% of the wild-type proportion of prestalk cells and did not differ significantly from each other (Table 1). When the mutants are allowed to develop in a 1:1 mixture, DIF-1 biosynthesis is restored by cross-feeding (Austin et al., 2006) and the wild-type number of prestalk cells recovered.

### Prestalk cell subtypes: des-methyl-DIF-1 is a repressor of prestalk-O differentiation

**Fig. 3:**
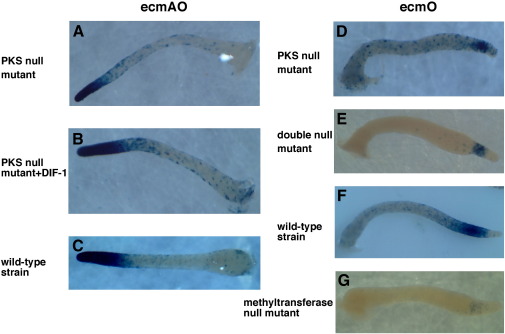
Prestalk subtypes in DIF-1 biosynthetic mutants. (A) ecmAO reporter expressed in the PKS null strain; (B) ecmAO reporter expressed in the PKS null strain with 100 nM DIF-1 added to the agar; (C) ecmAO reporter expressed in the wild-type strain; (D) ecmO reporter expressed in the PKS null strain; (E) ecmO reporter expressed in the PKS/methyltransferase double null strain; (F) ecmO reporter expressed in the wild-type strain; (G) ecmO reporter expressed in the methyltransferase null mutant. Reporters are as described (Jermyn et al., 1989; Early et al., 1993).

To try and pin down a more specific defect in prestalk cell differentiation, reporters derived from the promoter of the prestalk ecmA gene were used. The intact promoter drives lacZ expression in the entire anterior prestalk region, as well as in scattered anterior-like cells in the prespore region. The prestalk region is slightly shorter in the PKS null mutant, consistent with the reduced proportion of prestalk cells and previous findings for the methyltransferase null mutant. This defect is corrected by developing the mutant on DIF-1 agar (Figs. 3A–C).

The prestalk zone can be divided into an anterior pstA and a posterior pstO region by the expression of lacZ driven by subfragments of the ecmA promotor (referred to as ecmA and ecmO regions, with the entire promotor being ecmAO; Jermyn et al., 1989; Early et al., 1993). As expected from previous work (Thompson and Kay, 2000b), first fingers produced by PKS null cells have a normal pstA zone (not shown). In contrast, expression of the pstO marker is reduced, though some expression definitely remains (Fig. 3D). We find a slight expression of the pstO marker in a newly made reporter strain derived from the methyltransferase null, HM1030, differing from the original report (Thompson and Kay, 2000b), but this is clearly less than in the PKS null mutant (Fig. 3G).

**Fig. 4:**
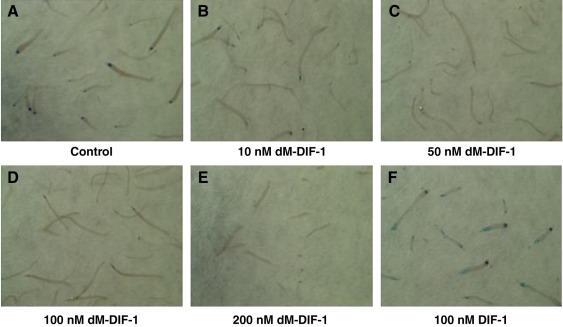
Des-methyl-DIF-1 represses prestalk-O cell differentiation. Low magnification images of ecmO-lacZ staining of double null mutant cells developed to the early slug stage with additions to the support filter as follows: (A) control, no additions; (B) 10 nM dM-DIF-1; (C) 50 nM dM-DIF-1; (D) 100 nM dM-DIF-1; (E) 200 nM dM-DIF-1; (F) 100 nM DIF-1. Approximately 50 nM dM-DIF-1 is required to suppress ecmO expression.

This unexpected difference in ecmO expression between the PKS and methyltransferase mutants could again be explained either by a build up of inhibitory biosynthetic intermediates in the methyltransferase mutant cells, or by a second function of DmtA in pstO cell differentiation. To discriminate between these possibilities, we examined the double null mutant, which lacks the biosynthetic intermediates. First fingers from this mutant were found to possess a pstO region resembling the PKS null mutant (Fig. 3E), suggesting that the blocked polyketide intermediates are actually inhibitory to pstO differentiation. We tested this idea further by developing the double null mutant on agar containing dM-DIF-1 and found that pstO differentiation is clearly inhibited, with an effect apparent at 50 nM dM-DIF-1 and complete inhibition by 100 nM dM-DIF-1 (Fig. 4).

### DIFless slugs lack a specific class of anterior-like cells

**Fig. 5:**
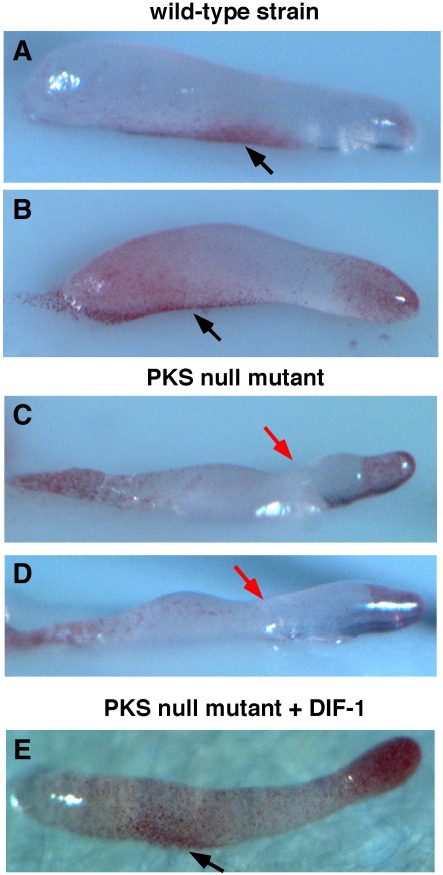
A class of anterior-like cells is lacking in the PKS null mutant. In the wild-type slugs, an aggregate of neutral red stained cells (indicated with black arrow) is located at the base of the prespore zone adjacent to the substratum (A and B). This aggregate of stained cells is absent in the PKS null mutant (C and D), but can be restored by development with DIF-1 (F). The dorsal side of the mutant slug is often bent, as in this case (indicated with red arrow in panels C and D).

Anterior-like cells are a heterogeneous class of prestalk cells that populate the prespore region of the slug and can be stained with the vital dye neutral red (Sternfeld and David, 1981). Figs. 5A and B show the typical side view of a wild-type slug stained with neutral red. The prestalk tip region is stained and there is also a notable aggregate of stained cells located in the prespore region adjacent to the substratum. These cells have been followed by time-lapse filming, and will later form the basal disc of the fruiting body (Dormann et al., 1996; Jermyn et al., 1996). Although PKS null slugs (taken very young before they break up) clearly have anterior-like cells, the aggregate adjacent to the substratum is missing, though it can be restored when the cells are developed on 100 nM DIF-1 (Fig. 5E). Furthermore, the mutant slugs are rather flat and the dorsal side is bent (Figs. 5C and D, red arrow).

### Fruiting body morphology: DIF-1 is required for basal disc formation and partially for lower cup formation

As the defects already noted in the final PKS null fruiting body (prostrate stalk and fallen sorus) are not obviously explained by the reduced number of prestalk cells, we examined the morphology of the fruiting body in more detail.

**Fig. 6:**
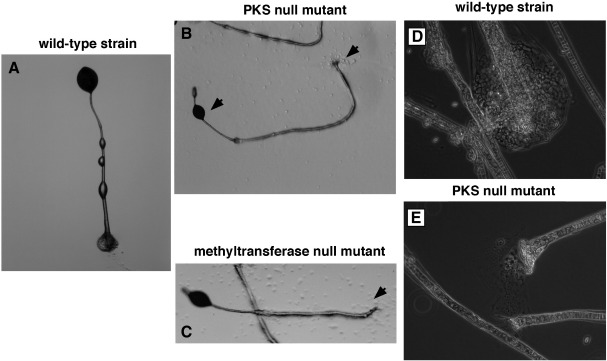
The fruiting bodies of the PKS null and methyltransferase null mutants lack a basal disc. Whole fruiting body structure of (A) wild-type strain; (B) PKS null mutant; (C) methyltransferase null mutant. The stalk of the PKS null mutant lies partially on the substratum and the spores slip down the stalk. Higher power detail of the basal discs of (D) wild-type strain; (E) PKS null mutant. The wild-type strain has both inner and outer basal discs, while the PKS null mutant has only the inner basal disc, which derives from the stalk proper.

The basal disc supports the stalk and forms from a separate group of anterior-like cells that gather where the base of the stalk will form, and eventually vacuolate (Dormann et al., 1996; Jermyn et al., 1996). The basal disc is almost entirely absent from the PKS null mutant fruiting bodies and the few that were scored as present were very defective, with the cells not necessarily vacuolated (Table 2, Fig. 6). A defect in basal disc formation was not previously reported in the methyltransferase null mutant fruiting bodies (Thompson and Kay, 2000b), but in fact it is quite apparent, although not as severe as in PKS null mutant fruiting bodies. Again it seems that this difference may be due to the presence of biosynthetic intermediates in methyltransferase null cells, as the double null mutant resembles the PKS null mutant in having essentially no basal discs (Table 2).

A more detailed examination showed that the PKS null mutant lacks the outer basal disc but retains the inner disc, which forms separately as a swelling from the base of the stalk (Figs. 6E and F). We also examined the fruiting bodies of the *dimA*^−^ and *mybE*^−^ DIF-insensitive mutants (Thompson et al., 2004; Fukuzawa et al., 2006). Plates of *mybE*^*-*^ fruiting bodies are very untidy, making them difficult to score, but we could not find any clear basal discs in this mutant. Only 4.5% of *dimA*^−^ fruiting bodies had a basal disc, compared to 90.4% for the Ax4 parent.

The spore mass is supported and capped by two cups of amoeboid cells, the upper and lower cups, which play a crucial role in raising and supporting the spores as they encapsulate and become immotile during culmination (Sternfeld, 1998). The upper and lower cup cells can be stained with neutral red: the upper cup is clearly present in PKS null mutants, but the lower cup is usually missing, while methyltransferase null mutants show a similar, but less severe effect.

**Fig. 7:**
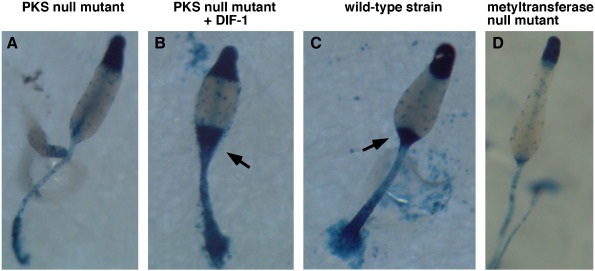
The lower cup is frequently missing in PKS null mutant fruiting bodies. Typical staining pattern of *ecmB*-lacZ in culminants: (A) PKS null mutant developed without DIF-1; (B) PKS null mutant developed with 100 nM DIF-1 added to the support filter (note the staining of the lower cup indicated by an arrow); (C) wild-type parental strain; (D) methyltransferase null mutant (note that there is some lower cup staining, but much less than the wild-type strain). Arrows indicate the lower cups.

We confirmed this using a lacZ marker driven by the *ecmB* promoter, which stains both the basal disc and lower cup in wild-type fruiting bodies (as well as the upper cup and stalk) but not in the PKS null mutant (Fig. 7). Again DIF-1 restores these structures. Quantitating this for the lower cup, 99% wild-type fruiting bodies had a stained lower cup and this was lacking in 74.7% of PKS null mutant culminants, but only 34.0% of methyltransferase null mutant culminates (Fig. 7) (Table 2).

## Discussion

The work described in this paper goes some way to describing the full complexity of the ‘DIFless’ mutant phenotype and hence the natural role of DIF-1 in development. It is centered on the phenotype of the PKS null mutant where the first step in DIF-1 biosynthesis is blocked, and supported by the methyltransferase null mutant phenotype, where the last step is blocked. The methyltransferase null mutant has been studied previously (Thompson and Kay, 2000b; Maeda et al., 2003; Maruo et al., 2004) but may give a less than complete phenotype because DIF-1 biosynthetic intermediates accumulate, which are active in stalk cell induction assays, and could therefore provide residual DIF activity (Saito et al., 2006).

### DIF-1 and slug migration

The lack of DIF-1 does not seem to affect the pacemaker region at the tip of the slug, which guides migration (Bretschneider et al., 1995), but somehow prevents the prespore region from responding efficiently, so that slug breaks up almost immediately on contacting the agar. It is unlikely that this is due to a mechanical weakness of the slime sheath, because the extra-cellular matrix proteins ecmA and ecmB are well-expressed in the mutant. More likely, it is due to the reduced efficiency of transmission of cyclic-AMP signals through the prestalk-O or prespore regions of the slug: one might speculate that anterior-like cells are crucial for signal transmission in the prespore region (Dormann et al., 2000). Since the isolated prespore regions reform a tip, it is also clear that prespore regulation does not depend on DIF-1.

### DIF-1 is required for proper basal disc and lower cup formation

The most striking feature of the PKS null mutant fruiting body is the near complete absence of a basal disc. This is a common feature of DIF-1 signaling mutants also found in *dmtA*, *dimA*, and *mybE* strains. In both PKS and methyltransferase null mutants, the defect was corrected by adding DIF-1 to the agar or by mixing the two mutants together to restore DIF-1 production (not shown). The lower cup of cells supporting the spore mass is also reduced or lost in the PKS null mutant, and to a lesser extent in the methyltransferase null mutant fruiting bodies. Thus, formation of the basal disc, and to a considerable extent the lower cup, depends on DIF-1.

The basal disc is a morphologically distinct part of the fruiting body, lending it stability. It forms from anterior-like cells in the prespore zone, not from the anterior prestalk zone (Raper, 1940; Sternfeld and David, 1982; Dormann et al., 1996; Jermyn et al., 1996) but the proportion of basal disc cells is regulated in parallel to the stalk proper (Stenhouse and Williams, 1977). Prebasal disc cells become immotile in a migrating slug at an early stage of culmination and act as a second signaling center, the tip being the first, which attracts anterior-like cells to the base of the aggregate (Dormann et al., 1996; Jermyn et al., 1996). Some of these form the basal disc, and the remainder the lower cup (which helps support the spore mass) indicating a close relationship between these two structures.

In terms of prestalk gene expression, basal disc and lower cup cells strongly express ecmA and ecmB, which are both inducible by DIF-1. The lower cup also expresses a marker gene of unknown function, *mrrA*, and this is dependent on MybE, which in turn is DIF-1-responsive (Tsujioka et al., 2007). In contrast, the cyclic-AMP receptor cAR2 (Saxe et al., 1996) and multi-drug resistance/serine protease homologue TagB (Shaulsky and Loomis, 1996), which are not inducible by DIF-1, are not expressed in the basal disc. TagB is expressed in the lower cup, suggesting that this tissue may be heterogeneous in origin. These marker experiments are therefore totally consistent with our finding that DIF-1 is required to induce the basal disc and partially for the lower cup.

The PKS null mutant fruiting body often lies with the lower part of its stalk prone on the agar, and frequently the spore mass slips down from the top of the stalk. Culmination of the mutant usually starts horizontally along the agar, and only after some stalk has been laid down does it turn upward, perhaps under the repulsive guidance of ammonia gas (Bonner and Dodd, 1962). We hypothesize that this behavior is due to the lack of prebasal disc cells, which appear to play a key role in triggering and perhaps orientating culmination (Dormann et al., 1996; Jermyn et al., 1996). The fallen spore mass is readily explained as due to the lack of an effective lower cup, which together with the upper cup supports the spore mass (Sternfeld, 1998).

Basal discs and related supporters are found in the fruiting bodies of only a minority of Dictyostelid species (Schaap et al., 2006). So far, DIF-1 is known to be made and metabolized by *D. mucoroides* (Kay et al., 1992; Van Es et al., 1994), which has a supporter rather than a basal disc, and it will be interesting to discover whether the presence of basal support in other species correlates with DIF-1 production or not.

### DIF-1, dM-DIF-1, and prestalk cell diversification

The number of types of prestalk cell is still debatable: classic studies from the Williams laboratory argue for three types in the anterior of the slug (prestalk-A, prestalk-O and prestalk-AB), plus an unknown number of types of anterior-like cells in the posterior (Williams, 1997). The wealth of gene expression patterns revealed by the new markers produced from microarray and *in situ* hybridization experiments suggests a much more complex reality (Maeda et al., 2003; Yamada et al., 2005). What is certain is that DIF-1 is only required for the induction of a subset of these prestalk cells, and that without DIF-1 there are 30–40% fewer prestalk cells (Thompson and Kay, 2000b; Maruo et al., 2004; this work). Our results indicate a specific loss of one class of anterior-like cells and of at least some pstO cells, but we cannot exclude a deficit in other prestalk cell types.

Our results unexpectedly suggest that desmethyl-DIF-1 (dM-DIF-1) has a role in cell-type proportioning. Genetically, the delayed tip formation by the methyltransferase null mutant and its reduced number of pstO cells compared to the PKS null mutant can be ascribed to a build up of dM-DIF-1, or possibly one of the other biosynthetic intermediates, and we find that dM-DIF-1 inhibits pstO cell differentiation in slugs. The same genetic argument also suggests that dM-DIF-1 can partially induce the anterior-like cells that form the basal disc. Earlier metabolic labeling experiments with ^36^Cl^−^ suggest that dM-DIF-1 is roughly equimolar with DIF-1 in pretip mounds (dM-DIF-1 is called ‘X’ in Fig. 2 of Kay, 1998), but then drops to barely detectable levels in slugs. It is thus possible that it plays a significant role at least in the mound stage of development.

The logic of prestalk cell diversification remains unclear. At one extreme, it might be proposed that each prestalk cell type has its own inducer, and cell type proportions are regulated by a feedback interaction with prespore cells as seen with DIF-1 (Insall et al., 1992; Kay and Thompson, 2001). In this case, the lack of one inducer, such as DIF-1, would result in the lack of a specific cell type, leaving the others unscathed. Or a more limited set of inducers might act in combination to specify a greater number of prestalk cells. In this case, lack of a specific inducer would affect several cell types. It is clear from the present work that the inducer of pstA cells at least lies outside the orbit of DIF-1, its precursors and metabolites, all of which are absent in the PKS null mutant we have studied. The identification of this inducer, which also seems to be a polyketide, is therefore a necessary step toward understanding the patterning in *Dictyostelium* (Serafimidis and Kay, 2005).

## Figures and Tables

**Table 1:** Strains used in this work

Strain	Stock Center ID	Relevant genotype	Phenotype	Referred to in text as	Reference
Ax2	DBS0235521	Wild-type	Wild-type	Wild-type	
HM1030	DBS0236259	*dmtA*^−^	dM-DIF-1 methyl transferase null	Methyl transferase null	Thompson and Kay, 2000b
HM1031		*dmtA*^−^	dM-DIF-1 methyl transferase null	Methyl transferase null	Thompson and Kay, 2000b
HM1154	DBS0236954	*stlB*^−^	SteelyB polyketide synthase null	PKS null	Austin et al., 2006
HM1196		*dmtA*^−^*, stlB*−	Double null	Double null	This work

All strains derive from the Kay laboratory version of Ax2, which is treated as the wild-type strain. HM1030 and HM1031 are independent *dmtA*^−^ isolates. Strains with a *Dictyostelium* Stock Center ID are available from: http://dictybase.org/StockCenter/StockCenter.html.

**Table 2:** Tip timing, prestalk cell proportioning and fate in DIF-1 biosynthetic mutants

Strain	Time to tip (h)	Proportion of prestalk cells	Basal disc (%)	Lower cup (%)
Ax2 wild-type	13.1 ± 0.3; 4	14.5 ± 1.0; 12	93.4 ± 1.1; 5	99.0 ± 0.5; 6
HM1030 *dmtA*	15.5 ± 0.6; 4	9.0 ± 0.6; 12	16.5 ± 2.7; 4	66.0 ± 4.8; 10
HM1031 *dmtA*	14.7 ± 0.5; 4	–	–	–
HM1154 *stlB*	12.5 ± 0.4; 3	9.9 ± 0.5; 10	5.4 ± 0.9; 4	25.3 ± 3.5; 8
HM1196 *dmtA/stlB*	12.7 ± 0.2; 4	–	1.4 ± 0.4; 5	4.8 ± 1.8; 8
HM1030/HM1154 1:1	–	13.6 ± 0.7; 9	–	–

Time to tip was determined as the time taken from the start of development for approximately half of the developing structures to form a morphological tip on triplicate plates for each experiment. The proportion of prestalk cells was determined in pools of around 20 individually collected first fingers by disaggregation and staining with an antibody that recognizes prespore cells. Unstained cells are assumed to be prestalk cells. Basal discs were scored microscopically on mature fruiting bodies; scoring was conservative, so that even a small gathering of cells at the base of a stalk was scored as a basal disc. The lower cup was recognized in mid-culminants of strains expressing ecmB-lacZ after lacZ staining. All strains produced a stalk and upper cup. Results are given with SEM and number of experiments; the difference in tip timing between Ax2 and HM1030 is significant (*p* < 0.05, Mann–Whitney two-tailed test), as are the differences in prestalk cell proportions between Ax2 and HM1030 and HM1154 (*p* < 0.01, Mann–Whitney two-tailed test).
