# Supplementary material for: DIF-1 induces the basal disc of the Dictyostelium fruiting body
Source: Dev Biol. 2008 May 15;317(2):444–53. doi: 10.1016/j.ydbio.2008.02.036 (PMC2726288; doi:10.1016/j.ydbio.2008.02.036)
Supplement: Supplementary Fig. 1 [file mmc1.pdf]

Supplementary information

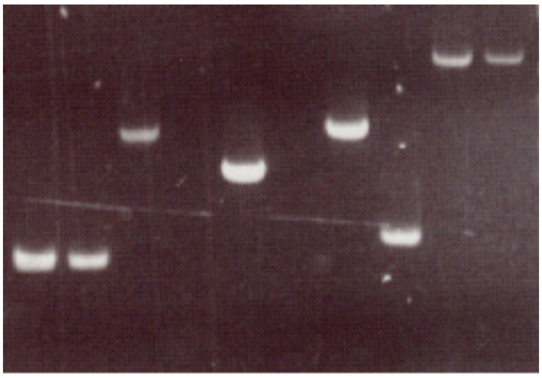

1 2 3 4 5 6 7 8 9 10

Figure 1. The disruptions of each gene locus were examined by genomic PCR. Primers dmtA-1 and dmtA-2 were used to confirm the disruption of the *dmtA* locus (lanes 1-3). The genomic DNAs of Ax2 (lane 1), HM1154 (lane 2), and HM1196 (lane 3) were used. HM1196 showed the insertion of basticidin resistance cassette and gave a product of about 2 kbp, while Ax2 and HM1154 gave products of about 700 bp. The primers II-5 and II-6 were used to confirm the disruption of the *stlB* locus (lane 4 and 5) of HM1154. II-6 is designed from the sequence of the blastcidin cassette and II-5 is derived from the *stlB* locus, 5' upstream of the knock out construct. Ax2 (lane 4) gave no band and HM1154 gave a 1.4 kbp PCR product (lane 5). Primers II-5 and II-9 were used to confirm the disruption of the *stlB* locus by the hygromycin resistance cassette (lanes 6 and 7) in HM1196. II-9 is designed from the sequence of the hygromycin resistance cassette. Ax2 (lane 6) gave no band and HM1196 (lane 7) gave a 2.0 kbp PCR product. Primers II-2 and II-3 were used to confirm the disruption of the *stlB* locus (lane 8-10). These primers were designed from *stlB* locus; Ax2 (lane 8) gave a 0.9 kbp band, while HM1154 (lane 9) and HM1196 (lane 10) gave about 3.9 kbp

PCR products.

primer	sequence
II-2	ATCATCACCAAATCCAATCGTTGTAC
II-3	AAATTACTGTTGCCATTGACGAATCG
II-5	TGGTGCTGAAATTCATGTTACAGTTGGTTC
II-6	GAGCCAATATGCGAGAACACCCGAGAA
II-9	GCCAACGACTACGCACTAGCCAAC
dmtA-1	TTGATGTTGCCATTGGGTATACAAAATC
dmtA-2	GTATAACAATCGGCAGAAGGATAATCAG
